# Mindfulness based cognitive therapy versus treatment as usual in adults with attention deficit hyperactivity disorder (ADHD)

**DOI:** 10.1186/s12888-015-0591-x

**Published:** 2015-09-15

**Authors:** Lotte Janssen, Cornelis C. Kan, Pieter J. Carpentier, Bram Sizoo, Sevket Hepark, Janneke Grutters, Rogier Donders, Jan K. Buitelaar, Anne E M Speckens

**Affiliations:** 1 Department of Psychiatry, Radboud University Medical Center, Nijmegen, The Netherlands; 2 Reinier van Arkel groep, ‘s-Hertogenbosch, The Netherlands; 3 Dimence, Deventer, The Netherlands; 4 Department for Health Evidence, Radboud University Medical Center, Nijmegen, The Netherlands; 5 Department of Cognitive Neuroscience, Radboud University Medical Center, Nijmegen, The Netherlands

## Abstract

**Background:**

Adults with attention deficit hyperactivity disorder (ADHD) often present with a lifelong pattern of core symptoms that is associated with impairments of functioning in daily life. This has a substantial personal and economic impact. In clinical practice there is a high need for additional or alternative interventions for existing treatments, usually consisting of pharmacotherapy and/or psycho-education. Although previous studies show preliminary evidence for the effectiveness of mindfulness-based interventions in reducing ADHD symptoms and improving executive functioning, these studies have methodological limitations. This study will take account of these limitations and will examine the effectiveness of Mindfulness Based Cognitive Therapy (MBCT) in further detail.

**Methods/design:**

A multi-centre, parallel-group, randomised controlled trial will be conducted in *N* = 120 adults with ADHD. Patients will be randomised to MBCT in addition to treatment as usual (TAU) or TAU alone. Assessments will take place at baseline and at three, six and nine months after baseline. Primary outcome measure will be severity of ADHD symptoms rated by a blinded clinician. Secondary outcome measures will be self-reported ADHD symptoms, executive functioning, mindfulness skills, self-compassion, positive mental health and general functioning. In addition, a cost-effectiveness analysis will be conducted.

**Discussion:**

This trial will offer valuable information about the clinical and cost-effectiveness of MBCT in addition to TAU compared to TAU alone in adults swith ADHD.

**Trial registration:**

ClinicalTrials.gov NCT02463396. Registered 8 June 2015.

## Background

Attention Deficit Hyperactivity Disorder (ADHD) is a common neurodevelopmental disorder with a high persistence into adulthood. Results of a meta-analysis of six studies indicated the pooled prevalence of adult ADHD according to DSM-IV criteria to be 2.5 % (95 % CI 2.1-3.1) [1]. In a Dutch population the prevalence of adult ADHD has been found to be 2.1 % (95 % CI 1.4-3.0) [2]. Patients with ADHD show a persistent pattern of inattention and/or hyperactivity-impulsivity, which leads to impairment of functioning in two or more areas in daily life, for example at work, school and/or home. These impairments have a substantial personal and economic impact. A recent study shows that ADHD is associated with increased mortality, mainly driven by deaths from unnatural causes like accidents [3]. Furthermore, adult ADHD is related to higher levels of unemployment and those who are employed often experience workplace impairment and reduced productivity [4]. In an international population ADHD is associated with 22 days of excess lost role performance among employees a year [5] and with elevated risk of high number of days out of role (OR = 2.6, 95 % CI 2.0-3.5) [6]. Results of a recent survey in a Dutch general population sample showed that adults with ADHD had significantly more functional impairments and an increased health care use than persons without ADHD [2].

The vast majority of ADHD patients in the Netherlands is offered stimulant medication after or parallel to psycho-education on ADHD. In accordance with the NICE guideline on ADHD [7] stimulants are the first-line treatment for adults as part of a comprehensive treatment programme that addresses psychological, behavioural and educational or occupational needs. The pooled effect size of stimulant medication in reducing ADHD symptoms was in the medium-to-high range [8]. Stimulants demonstrated efficacy and they seemed to improve work productivity [9]. Moreover drug treatment in ADHD seemed to have a protective effect on the risk of criminality [10] and suicidal behaviour [11]. Although pharmacological treatment has thus shown to be effective in reducing ADHD symptoms and to have beneficial effects, about 10-30 % of adults with ADHD do not adequately respond to stimulants [12] and often report adverse effects like decreased appetite, dry mouth, tension or jitteriness [13], which can lead to discontinuation. It has also been found that these patients were compliant with medication for rather a brief period (M = 49.5 days), and the average persistence with medication was less than a year (M = 199.9 days) [14]. Besides, some patients with ADHD are not willing to take medication at all. Furthermore, the evidence for long-term effects of stimulants is sparse [15]. Therefore, there is a strong need for evidence-based psychosocial interventions, as an addition to or an alternative for pharmacological treatment of adult ADHD.

The NICE guideline recommends group Cognitive Behavioural Therapy (CBT) for adults with ADHD whose response to medication is insufficient, who have difficulty adhering to drug treatment or who are not willing to use medication. Some studies have shown evidence for the efficacy of treatment with CBT in adults with ADHD, particularly when combined with medication [16]. For example, Safren et al. [17] found support for the effectiveness of individual CBT for patients with ADHD, who are treated with medication but continue to have residual symptoms, compared to a time-matched comparison treatment. Another study of Solanto et al. [18] showed that meta-cognitive group therapy, which is a cognitive-behavioural intervention, provides significant benefit to patients with ADHD with respect to the severity of ADHD symptoms in comparison with supportive group therapy. However, more controlled research studies are required. Another promising psychosocial intervention for patients with ADHD is Mindfulness-Based Cognitive Therapy (MBCT), which combines methods of CBT and mindfulness meditation. Mindfulness is defined as paying attention in a particular way: on purpose, in the present moment, and non-judgmentally [19]. MBCT is usually offered in a group format, consisting of 8 weekly sessions of 2,5 hours and a silent day [20], which may be more cost-effective than individual treatment. In recent years, mindfulness based therapy, such as MBCT, has been demonstrated to reduce anxiety, depression and stress in a wide range of psychiatric disorders [21], including current anxiety and depressive disorders [22]. Furthermore, there is evidence that mindfulness meditation strengthens attention regulation [23–25] and improves some executive functions [26] in healthy subjects. Although these studies were not focused on patients with ADHD and replication studies need to be carried out, this is promising evidence in the search for an effective non-pharmacological treatment for this patient group.

A few studies on the effectiveness of mindfulness training for adults with ADHD have so far been conducted, two uncontrolled pilot studies [27, 28] and two randomised controlled trials (Hepark, Janssen, De Vries, Schoenberg, Kan, & Speckens: The effects of Mindfulness-Based Cognitive Therapy on core sympotms and executive functioning in adults with ADHD: a preliminary randomised controlled trial, submitted) [29]. The studies of Zylowska et al. [27] and Mitchell et al. [29] used the 8-week Mindful Awareness Practices (MAPs) for ADHD programme [30], while our own group [28] (Hepark et al., submitted) used an adapted and extended MBCT programme. All of these studies observed a substantial reduction of primary ADHD symptoms, and the effect of mindfulness training on ADHD symptoms seems to be mediated by an increase of mindfulness skills (Hepark et al., submitted). Moreover, improvements of executive functioning (Hepark et al., submitted) [29] and emotion regulation [29] were reported. In both pilot studies [27, 28] improved executive control was observed using neuropsychological tasks, but this was not replicated in the two randomised controlled trials. Empirical evidence regarding the reduction of secondary symptoms, like depressive and anxiety symptoms and general functioning, is mixed [27, 28] and (Hepark et al., submitted). Possibly, this is partially due to baseline differences in levels of secondary symptoms in the various samples.

The results of the above mentioned studies support the feasibility and efficacy of mindfulness training as a treatment for adults with ADHD and encourage further research. This approach offers a novel and potentially useful tool in the multimodal treatment of ADHD. Previous studies are conducted in one centre and lack a follow-up period. Some of them are small [27–29], uncontrolled [27, 28] and lack blinding [27–29]. The proposed study is the first properly powered randomised controlled multi-centre study, which includes blinded outcome assessments, a longer term follow-up period and assessment of cost-effectiveness.

### Aims of the trial

The primary aim of this study is to investigate effectiveness of MBCT in addition to Treatment As Usual (TAU) versus TAU alone in adults with ADHD. We expect that MBCT can improve ADHD symptoms and overall functioning in two ways: a) by strengthening their attention regulation and executive functioning, resulting in a greater reduction of ADHD symptoms rated by a blinded clinician (primary outcome) and self-reported ADHD symptoms and a greater improvement of executive functioning (secondary outcomes); and b) by extending their repertoire of coping strategies to deal with core symptoms and functional impairments, resulting in a greater improvement of general functioning, positive mental health and self-compassion (secondary outcomes).

Other aims of the study are to assess whether MBCT is a cost-effective intervention compared to TAU in patients with ADHD from a societal perspective and to assess the financial consequences of implementing mindfulness training in patients with ADHD from different perspectives (e.g. societal, health care, health insurance and patient). We expect that the implementation of this intervention will result in a reduction of medical and societal costs in the MBCT group compared to the TAU group.

Furthermore, in order to gain insight in the working mechanisms of mindfulness, we will investigate whether possible clinical effects of MBCT can be attributed to changes in mindfulness skills, self-compassion and adherence to MBCT.

## Methods/design

### Study design

The design of the study is multi-centre, parallel-group, randomised controlled superiority trial. Patients will be randomised to MBCT in addition to TAU or TAU alone. The study protocol has been ethically approved by CMO Arnhem-Nijmegen for all participating centres and registered under number 2014/206.

### Participants

The study population will consist of adults with ADHD. To enhance generalisability, the study is conducted in three different mental health centres for patients with ADHD in the south-east of the Netherlands: the Department of Psychiatry of the Radboudumc in Nijmegen, the Reinier van Arkel Group in the Jeroen Bosch hospital in ‘s Hertogenbosch and Dimence in Deventer. Each centre has a specialized outpatient clinic for adults with developmental disorders, like ADHD.

#### Eligibility criteria

We will include patients of 18 years and older who meet the following criteria: a) a primary diagnosis of ADHD, according to the criteria of Diagnostic and Statistical Manual of Mental Disorders – 4^th^ edition (DSM-IV-TR) [31] based on a structured Diagnostic Interview for ADHD, in adults (DIVA) [32]; and b) capable of filling out questionnaires in Dutch. Exclusion criteria will be: a) depressive disorder with psychotic symptoms or suicidality; b) current manic episode; c) borderline or antisocial personality disorder; d) substance dependence; e) autism spectrum disorder; f) tic disorder with vocal tics; g) learning difficulties or other cognitive impairments; and h) former participation in a MBCT or MBSR course or workshop of more than two hours’ duration. We will use a psychiatric structured diagnostic interview (MINI-Plus) [33] to establish whether patients meet the above mentioned exclusion criteria a, b and d. To assess the third exclusion criterion, screening lists for borderline and antisocial personality disorders based on the Structured Clinical Interview for DSM-IV Axis II Disorders (SCID-II) [34] will be administered. In patients meeting five or more criteria on one or both of the lists, the entire interview for borderline and/or antisocial personality disorder of the SCID-II will be conducted.

### Procedure and treatment allocation

Recruitment will take place in different ways: by referral via the specialist outpatient clinics of the three participating centres and by self-selection. Currently and formerly treated patients with ADHD at the Radboudumc, the Reinier van Arkel Groep and Dimence will be informed about the study by their attending clinicians in various stages of their treatment process. Patients will also be recruited by self-selection by media advertisements, for example through the website of the Dutch association of adults with ADHD (Impuls & Woortblind) and in presentations at regional thematic meetings. Interested patients will receive an information leaflet and are invited for a screening by telephone with the researcher or research assistant to assess eligibility. If patients seem to be eligible, they will get one to two weeks time to consider participation. Patients who chose to participate will be invited for an interview with the researcher or research assistant in which written informed consent will be obtained.

When the Diagnostic Interview for ADHD in adults (DIVA) has not been conducted to diagnose ADHD before, it will be administered by a psychologist or psychiatrist with relevant experience with this specific patient group to check whether a patient indeed meets the DSM-IV criteria for ADHD. Whenever possible the interview will take place in the presence of a family-member, partner or significant other to enable retrospective and collateral information to be ascertained simultaneously.

Blinded assessments with the investigator-rated screening version of the Conners’ Adult ADHD Rating Scale (CAARS-INV) will be obtained by trained psychiatrists or specialist registered nurses (in training) at baseline, 3, 6 and 9 months after baseline. The 6 and 9 months follow-up assessments will take place by phone. Self-report questionnaires will be administered at all of the above mentioned four time points. In order not to disadvantage patients randomised to the TAU group, we will offer them MBCT after completion of the study at 9 months follow-up.

**Fig. 1:**
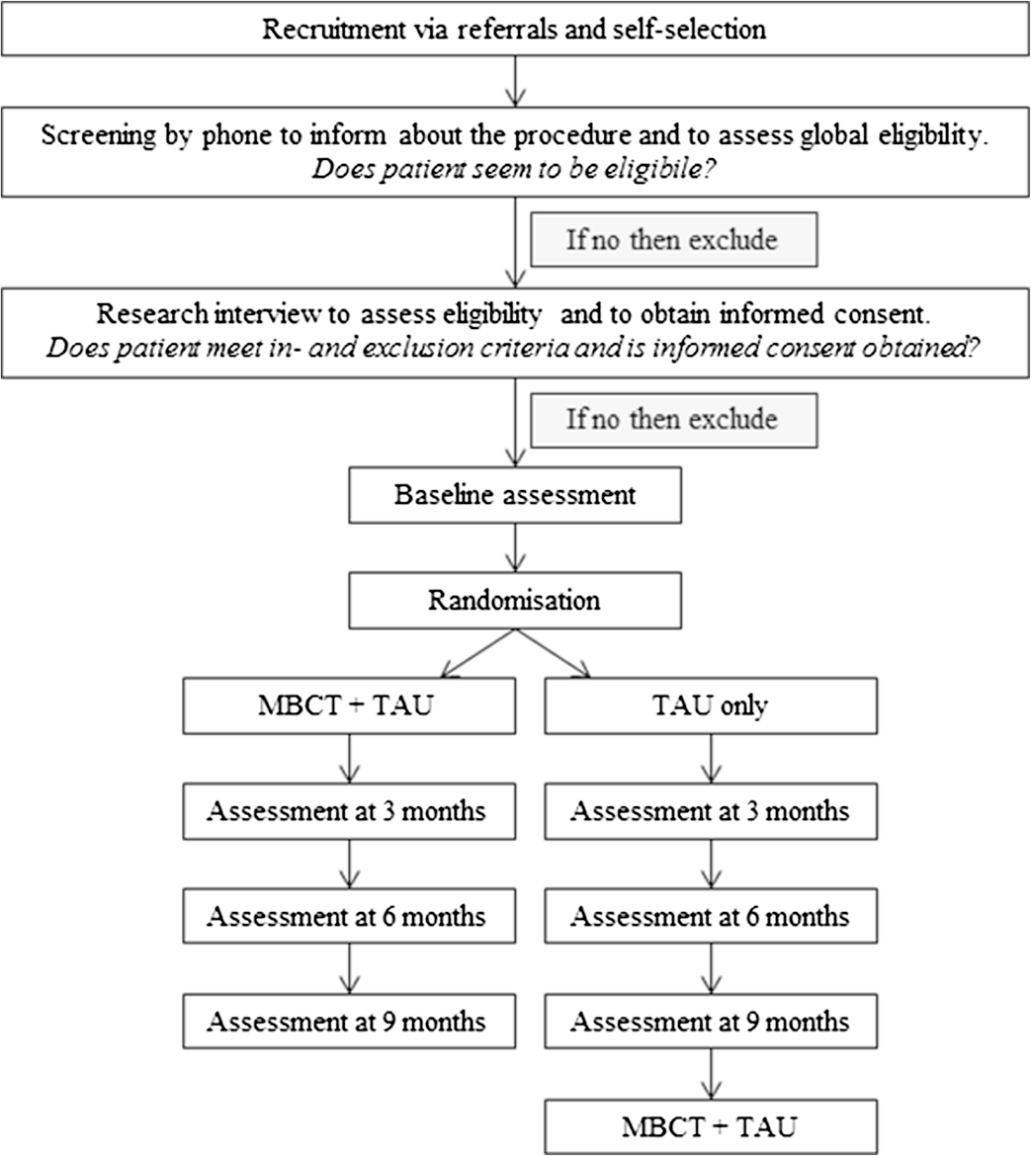
Of the recruitment and study procedure

Patients will be able to use medication for ADHD such as psycho-stimulants, during the entire study and they will not be withheld from other psychosocial treatments they might be offered. Concurrent interventions will be closely monitored and registered. See Fig. 1 for a flowchart of the recruitment and study procedure.

### Randomisation

Randomisation will be computerised and be stratified for centre. Block randomisation with varying predefined block sizes is used combined with minimisation. Block randomisation will be dominant over minimisation. The randomisation will be minimised taking account of: a) use of medication for ADHD (medication versus no medication); b) past participation in a psycho-education training (psycho-education versus no psycho-education); c) gender (male versus female); and d) subtype ADHD (combined, hyperactive-impulsive, inattentive and not otherwise specified). The researcher will be blind for the block sizes.

### Blinding

Blinded assessments by a psychiatrist or specialist nurse with the investigator-rated screening version of the Conners’ Adult ADHD Rating Scale (CAARS-INV) will take place at baseline and 3, 6 and 9 months after baseline. At baseline patient nor interviewer will know in which group the patient will be placed. To ensure that the interviewers stay blind during the other time points of assessment, patients will be instructed at the research interview not to share information about allocation with the interviewer. At the beginning of each assessment, the interviewer will repeat this instruction.

### Outcome measures

#### Selection and diagnostic measures

The Diagnostic Interview for ADHD in adults (DIVA) [32] is a structured interview used in our study to diagnose ADHD in adults. The first part consists of the DSM-IV criteria of ADHD in childhood and adulthood and the second part is to determine impairment in five domains (education and work, relationships and family life, social contacts, leisure time and self-confidence) in both life periods. Whenever possible, the information provided by the patient is supplemented by collateral information from a family member or partner and by school reports.

#### Primary outcome measure

##### ADHD symptoms (investigator-rated)

We will use the investigator-rated screening version of the Conners’ Adult ADHD Rating Scale (CAARS-INV: SV) [35] by a blinded clinician to assess DSM-IV criteria symptoms as well as specific manifestations of ADHD, such as mood lability. This interview consists of 30 items, which are rated on a 4-point Likert scale. Ratings can be organised in three subscales: Inattention, Hyperactivity/Impulsivity, and the ADHD index. All subscales have shown good internal consistency and inter-rater reliability, as well as sensitivity to treatment outcome [36]. Cronbach’s α coefficients vary from .74 to .94 and intraclass correlations between investigator-ratings and self-ratings ranged from .45 to .87 [36].

#### Secondary outcome measures

##### ADHD symptoms (self-reported)

The self-report version of the Conners’ Adult ADHD Rating Scale (CAARS-S:SV) [35] will be used to assess self-reported ADHD symptoms. This questionnaire consists of 30 items, which are rated on a 4-point Likert scale, and has the same structure as the investigator version. The internal consistency is comparable with that of the investigator version and Cronbach’s α coefficients vary from .76 to .95 at different time points [36].

##### Executive functioning

The self-report version for adults of the Behaviour Rating Inventory of Executive Function (BRIEF-A) [37] will be used to measure executive functioning in daily life. This questionnaire consists of 75 items on a 3-point Likert scale and has nine subscales: Inhibit, Shift, Emotional Control, Self-monitor, Initiate, Working Memory, Plan/Organise, Task Monitor, and Organization of Materials. Three validity scales are included: Negativity, Infrequency, and Inconsistency. For the Dutch version of the BRIEF-A internal consistency varies with Cronbach’s α coefficients ranging from .70 to .89 and test-retest reliability was found to be .70 or higher [38].

##### Mindfulness skills

We will use the short form of the Five Facet Mindfulness Questionnaire (FFMQ-SF) [39, 40] to measure mindfulness skills. This self-report questionnaire consists of 24 items which are rated on a 5-point Likert scale. Five facets of mindfulness are distinguished: Observing, Describing, Acting with Awareness, Non-judging, and Non-reactivity. The Dutch version of the FFMQ-SF has been shown to be a reliable instrument which is sensitive to change in a population with depressive and anxiety symptoms. Cronbach’s α coefficients between .75 and .87 have been found [39].

##### Self-compassion

The short form of the Self-Compassion Scale (SCS-SF) [41, 42] will be used to measure self-compassion. This instrument consists of 12 item based on a 7-point Likert scale and measures six components of self-compassion: Self-kindness, Self-judgement, Common Humanity, Isolation, Mindfulness and Over-identification. The shortened scale shows a near-perfect correlation with the original Self-Compassion Scale. In a Dutch population, Cronbach’s α coefficient for the full scale is .87 and the coefficients for the subscales of the SCS-SF vary from .55 to .81. The internal consistency of one of the components (Self-kindness) was relatively low [41].

##### Positive mental health

The short version of the Mental Health Continuum (MHC-SF) will be used to measure positive mental health [43, 44]. This self-report questionnaire has 14 items which are scored on a 6-point Likert scale and three subscales: Emotional, Psychological and Social. The Dutch version of this instrument has a good internal reliability with Cronbach’s α coefficients varying between .74 and .89 [43].

##### General functioning

We will use the Outcome Questionnaire (OQ 45.2) [45] to measure general functioning. This self-report questionnaire consists of 45 items and three subscales: Symptom Distress, Interpersonal Relations, and Social Role. The items are scored on a 5-point Likert scale. Cronbach’s α coefficients ranged from .70 to .93 and test-retest reliability with Pearson’s *r* varied from .78 to .84 [45]. The Dutch version of the OQ has comparable psychometric properties with Cronbach’s α coefficients between .69 and .93 and Pearson’s *r* from .70 to .83 in a clinical population [46].

#### Cost-effectiveness

##### Resource use

Resource use will be collected and calculated with the latest version of the Trimbos/iMTA questionnaire for costs associated with psychiatric illness (TiC-P) [47]. This self-report instrument consists of two parts: a) healthcare consumption including contacts within the mental and the general healthcare sector and the use of medication; b) productivity loss. The recall period for the TiC-P is three months for healthcare consumption and one month for productivity loss. Test-retest reliability is satisfactory to almost perfect for most items related to healthcare consumption and satisfactory for items on absence from work and presenteeism. Cohen’s kappa values range from .49 to .84 [48].

##### Quality Adjusted Life Years (QALYs) health gains

We will use the 12-item Short Form (SF-12) Health Survey [49], a shortened version of the SF-36 [50], which is a widely used instrument for measuring health status to calculate QALYs. The SF-12 is a self-report questionnaire with eight domains divided in two components: a physical component summary (Physical Functioning, Role Limitations due to Physical Problems, Bodily pain, General Health) and a mental component summary (Role Limitations due to Emotional Problems, Mental Health, Social Functioning, Vitality). Acceptable levels of internal consistency have been found, with Cronbach’s α coefficients of .84 for the physical component and .81 for the mental component [51]. Test-retest reliability coefficients of .89 for the physical component and .76 for the mental component have been measured in a US population [49].

#### Process measure

##### Adherence to MBCT

During the nine weeks of participation in the MBCT group, we will assess adherence to mindfulness exercises with a calendar on which patients fill out total minutes of formal (e.g. sitting meditation) and informal practice (e.g. mindful eating) on a daily basis. For patients allocated to the MBCT group, we will assess adherence during 6 and 9 months after baseline by questions in which patients are asked to estimate total minutes of formal and informal practice during an average week in the last three months. To check for compliance with the instruction to refrain from mindfulness training during the nine months of the study, patients in the TAU group will be asked whether they participated in an MBSR or MBCT course during the study at the different time points of assessment.

### Intervention

We developed a protocol primarily based on the Mindfulness-Based Cognitive Therapy (MBCT) for recurrent depression [20]. We made some modifications to make the intervention more suitable for adults with ADHD. For example, the psycho-education sections about depression are replaced by psycho-education about ADHD. We also include elements of the Mindful Awareness Practices for ADHD programme (MAPs) [30]. For example, in accordance with the MAPs protocol: a) the meditation periods are built up more gradually compared to the regular MBCT programme; b) mindful awareness in daily life is emphasized; and c) one session is focused on mindful listening and speaking. Table 1 shows the content of the MBCT programme as it will be used.

**Table 1:** Content of MBCT programme for ADHD per session

Theme of the session	Mindfulness exercises	(Psycho-)education	Homework
1. Automatic pilot	- 3-min breathing space	- Rationale of mindfulness for ADHD	- 3-min breathing space
	- Raisin exercise	- Introduction in methods to integrate mindfulness in daily life: Support, Structure and Strategy (3 S’s)	- Attention for a routine activity
	- Bodyscan		- Mindful eating
			- Optional: bodyscan
2. Dealing with barriers	- Bodyscan	- Imagery exercise to demonstrate relationship between thoughts and feelings	- 3-min breathing space
			- Attention for a routine activity
			- Bodyscan
	- Sitting meditation with focus on breath	- Exploration of application of the 3 S’s	- Awareness of pleasant events
	- 3-min breathing space		
3. Mindfulness of the breath	- Sitting meditation with focus on breath, body	- Seeing exercise a) to demonstrate the difference between observation and interpretation, b) to discuss dealing with sensory input	- 3-min breathing space
			- Floor yoga or sitting meditation
	- 3-min breathing space	- Exploration of pleasant events	- Awareness of unpleasant events
	- Floor yoga practices		
	- Walking meditation		
4. Staying present	- Sitting meditation with focus on breath, body, sounds	- Exploration of unpleasant events with attention for the interrelatedness of feelings, thoughts and bodily sensations	- 3-min breathing space
			- Sitting meditation
	- 3-min breathing space	- Exercise focused on recognition and dealing with ADHD core symptoms	- Awareness of top 3 ADHD symptoms
	- Walking meditation		
5. Allowing and letting be	- Sitting meditation with focus on breath, body, sounds, thoughts and feelings	- Reflection on intention of participating	- 3-min breathing space
		- Psycho-education about reacting versus responding in stressful situations and when ADHD symptoms are severe	- Sitting meditation or standing yoga
	- 3-min breathing space ‘coping’		- Awareness of communication difficulties
	- Standing yoga practices		
6. Mindful communication	- Standing yoga practices	- Exercise in mindful listening and speaking	- 3-min breathing space
	- 3-min breathing space	- Non-verbal communication exercise	- Sitting meditation or standing yoga
			- Mindful listening and speaking
Silent day	- Varying meditation exercises		
	- Silent lunch and tea break		
7. Taking care of yourself	- Sitting meditation with focus on breath, body, sounds, thoughts, emotions an choiceless awareness	- Exercise on taking care of yourself by examining how to improve balance in life	- 3-min breathing space
			- Practice without CDs
			- Reflect on training
	- 3-min breathing space		- Making an action plan
8. The rest of your life	- Bodyscan	- Reflection on the training	
	- 3-min breathing space	- Maintaining practice	

The protocol has been tested in three consecutive pilot groups of ADHD patients and has been adapted according to their feedback. The 8-week programme will consist of weekly sessions lasting 2.5 hours, and a silent day between session six and seven. In addition to the group sessions, participants will be instructed to practice six days a week for approximately 30 min a day and complete home practice assignments. The participants will receive a set of CDs containing guided mindfulness exercises for home practice ranging in length from 10 min to 45 min.

#### Mindfulness teachers

The MBCT will be taught by mindfulness teachers meeting the advanced criteria of the Association of Mindfulness Based Teachers in the Netherlands and Flanders and the internationally agreed good practice guidelines of the UK Network for Mindfulness-Based Teachers http://mindfulnessteachersuk.org.uk/pdf/teacher-guidelines-2015.pdf) which include: a) a minimum of 150 h of education in MBSR/MBCT background and theory including a reflection report; b) relevant professional training; c) minimum of three years of practicing meditation regularly and attending retreats; d) having attended MBSR/MBCT as a participant; e) continued training; and f) giving a minimum of two courses per two year. The teachers will receive additional training in the study protocol at the start of the project. We will organise peer supervision meetings every three weeks during the intervention phase of the trial. Intervention integrity consisting of adherence to the original programme, the degree to which the intervention is delivered as intended and teacher competency, will be assessed by the Mindfulness-based Interventions-Teaching Assessment Criteria (MBI: TAC) [52]. Videotapes of a random selection of sessions will be assessed by assessors who are familiar with this mindfulness-based programme, are 'proficient' (level 5) according to the MBI: TAC and have received training in the use of these assessment criteria.

### Sample size

The power calculation has been based on our previous randomised controlled study consisting of 103 adults with ADHD (Hepark et al., submitted) in which we demonstrated a significant difference in post-assessment clinician-rated ADHD symptoms (CAARS-INV) [36] between the MBCT and waitlist control group. Based on these study results, we expect a post-treatment difference of 4 between the two groups with a standard deviation of 7.5. Using a two-sided test with an alpha of 0.05, a power of 80 % and analyzing the data using ANCOVA assuming the correlation between pre- and post-assessments to be 0.5, we would need approximately 45 patients per arm. Taking account for loss of power due to drop out of 25 %, we intend to include 120 patients, 60 patients per group.

### Statistical analyses

#### Primary analyses

Baseline socio-demographic and clinical characteristics of the MBCT group and the TAU group will be compared to check whether these characteristics will have been evenly distributed by randomisation. The primary analyses will be aimed at comparing results on the outcome measures at three months after baseline between the MBCT group and the TAU group, controlling for baseline levels and possible other baseline differences between intervention and control groups (despite randomisation).

The outcome data will be analysed and reported according to the CONSORT guidelines and will include both intention-to-treat analyses and per-protocol analyses with the treatment-adherent sample (patients in the MBCT group have to attend at least four of MBCT sessions and patients in the TAU group do not attend a mindfulness-based programme). Percentages of improved, recovered, unchanged and worsened patients in the intervention and control groups will be provided on the basis of predefined cut-off scores (*T-*score greater than 65) and standard deviations. In addition, a Cohen’s *d*-type effect size will be calculated.

In order to investigate consolidation of treatment effect at 6 and 9 months after baseline, we will study whether there is an intervention by time interaction with a mixed model to accommodate for repeated measures. Such an interaction would imply that long term effects are different from short term effects. In addition, regression models will be used to derive prediction models for therapy outcome.

#### Secondary analyses

##### Cost-effectiveness analysis

For each patient in the MBCT and TAU group: a) costs; b) treatment response; and c) QALYs will be collected over the study period of nine months. Total costs for each patient will be obtained by multiplying the resource use as measured with the TiC-P with standard costs, based on the Dutch guideline for costing research [53]. Overall mean and median costs will be compared across the two groups and where relevant, differences will be calculated inclusive of 95 % confidence intervals. Treatment response will be based on our primary outcome, the investigator-rated version of the CAARS. QALYs will be assessed by the SF-12. At baseline and each follow-up interval, each patient is assigned a utility score based on the SF-12. From these utility scores QALYs will be calculated for each patient using the Area Under the Curve (AUC) method. Mean and median QALYs will be compared across the two groups and where relevant, differences are calculated inclusive of 95 % confidence intervals.

If MBCT is more costly and more effective than TAU, an incremental cost-effectiveness ratio (ICER) will be calculated by dividing the extra costs by the extra effects. ICERs are computed to obtain the costs per responder and the costs per QALY gained. This will give an estimation of the extra costs that are needed to gain one responder or QALY. If this is below the societal willingness to pay, mindfulness training is deemed cost-effective. If one comparator is less costly and more effective, it dominates the other and no ICER is necessary to determine cost-effectiveness. If MBCT is less effective and less costly, ICERs will be calculated representing the savings per QALY lost. Uncertainty surrounding the costs, effects and ICER will be addressed by means of bootstrapping.

##### Mediation analysis

To gain insight in underlying working mechanism of mindfulness, we will test with a mediation analysis whether possible clinical effects of MBCT can be attributed to changes in mindfulness skills, self-compassion and adherence to MBCT. We will follow the approach suggested by Preacher, Hayes [54].

## Discussion

Adult ADHD has a substantial personal and economic impact and consequently there is a high need for evidence-based treatments for this patient group. Although pharmacotherapy has shown to be effective in reducing ADHD symptoms and its economic impact, there is a call for psychosocial interventions to offer in addition to pharmacotherapy or as an alternative treatment. At this moment research examining the effectiveness of psychosocial interventions for adult ADHD is still in its infancy. Mindfulness training, such as MBCT, is a promising psychosocial intervention, which can be offered in a group format. Considered from an economic perspective, this is an advantage over treatment on individual basis. Previous studies support the feasibility of mindfulness training for this patient group and provide preliminary, but consistent evidence that this intervention reduces ADHD symptoms and improves executive functioning. However, the methodology of these studies is limited by single-centre enrolment [27–29], and (Hepark et al., submitted) lack of follow-up periods [27–29], and (Hepark et al., submitted) small sample sizes [27–29], lack of a control group [27, 28] and lack of blinded assessments [27–29]. Our study will take account of these limitations and will examine the effectiveness of MBCT in adults with ADHD in further detail.

To our knowledge, this is the first properly powered randomised controlled study in this field, that contains blinded outcome assessments and an intention-to-treat analysis. Besides, a follow-up period with a length of six months after the end of treatment is included to consider potential long-term effects of MBCT. To enhance generalisability, this study is conducted in three different mental health centres with expertise in treating adults with ADHD. Additionally, cost-effectiveness will be assessed to evaluate whether MBCT is a cost-effective intervention compared to the treatment as usual and to examine the financial consequences of implementing this intervention. This trial will offer valuable information on whether MBCT has an effect on clinician-rated and self-reported ADHD symptoms, executive functioning, general functioning, positive mental health, self-compassion and medical and societal costs in comparison to treatment as usual.

## Abbreviations

ADHD: Attention deficit hyperactivity disorder
ANCOVA: Analysis of covariance
AUC: Area under curve
BRIEF-A: Behaviour rating inventory of executive function-adult
CAARS: Conners adult ADHD scale
CBT: Cognitive behavioural therapy
CMO: Central committee on research involving human subjects
CI: Confidence interval
CONSORT: Consolidated standards of reporting trials
DIVA: Diagnostic interview for ADHD in adults
DSM-IV: Diagnostic and statistical manual of mental disorders 4^th^ edition
FFMQ-SF: Five facet of mindfulness questionnaire-short form
ICER: Incremental cost-effectiveness ratio
INV: Investigator
ISPOR: International society for pharmacoeconomics and outcomes research
MAPs: Mindful awareness practices
MBCT: Mindfulness-based cognitive therapy
MBI:TAC: Mindfulness based interventions-teacher rating scale
MBSR: Mindfulness based stress reduction
MHC-SF: Mental health continuum-short form
MINI: Mini international neuropsychiatric interview
NICE: National institute for health and care excellence
OQ: Outcome questionnaire
SCID-II: Structured clinical interview for DSM-IV axis II disorders
SCS-SF: Self-compassion scale-short form
SF-12: Medical outcomes study 12-item short form health survey
SV: Screening version
TAU: Treatment as usual
TiC-P: Trimbos institute and iMTA questionnaire on costs associated with psychiatric illnesses
QALY: Quality adjusted life year
